# Normative study of SATURN: a digital, self-administered, open-source cognitive assessment tool for Italians aged 50–80

**DOI:** 10.3389/fpsyg.2024.1456619

**Published:** 2024-10-30

**Authors:** Francesco Giaquinto, Sara Assecondi, Giuliana Leccese, Daniele Luigi Romano, Paola Angelelli

**Affiliations:** ^1^Laboratory of Applied Psychology and Intervention, Department of Human and Social Sciences, University of Salento, Lecce, Italy; ^2^Center for Mind/Brain Sciences – CIMeC, University of Trento, Rovereto, Italy; ^3^Laboratory of Applied Psychology and Intervention, Department of Medicine, University of Salento, Lecce, Italy; ^4^Department of Psychology and Milan Center for Neuroscience (NeuroMi), University of Milano-Bicocca, Milan, Italy

**Keywords:** cognitive assessment, self-administered cognitive test, computer based test, aging, normative data

## Abstract

**Introduction:**

This study aimed to establish normative data for the Self-Administered Tasks Uncovering Risk of Neurodegeneration (SATURN), a brief computer-based test for global cognitive assessment through accuracy and response times on tasks related to memory, attention, temporal orientation, visuo-constructional abilities, math (calculation), executive functions, and reading speed.

**Methods:**

A sample of 323 Italian individuals with Montreal Cognitive Assessment (MoCA) equivalent score ≥1 (180 females; average age: 61.33 years; average education: 11.32 years), stratified by age, education, and sex, completed SATURN using PsychoPy, and a paper-and-pencil protocol consisting of Mini-Mental State Examination (MMSE) and MoCA. Data analyses included: (i) correlations between the total accuracy scores of SATURN and those of MMSE and MoCA; (ii) multiple regressions to determine the impact of sex, age, and education, along with the computation of adjusted scores; (iii) the calculation of inner and outer tolerance limits, equivalent scores, and the development of correction grids.

**Results:**

The mean total time on tasks was 6.72 ± 3.24 min. Age and education significantly influence the SATURN total accuracy, while sex influences the total time on tasks. Specific sociodemographic characteristics influence subdomain accuracies and times on task differently. For the adjusted SATURN total score, the outer limit corresponds to 16.56 out of 29.00 (cut-off), while the inner limit is 18.57. SATURN significantly correlates with MMSE and MoCA.

**Discussion:**

In conclusion, SATURN is the first open-source digital tool for initial cognitive assessment in Italy, showing potential for self-administration in primary care, and remote administration. Future studies need to assess its sensitivity and specificity in detecting pathological cognitive decline.

## 1 Introduction

As individuals age, age-related cognitive decline becomes more common. The prevalence of cognitive difficulties in Europe varied from 13.0 to 50.5%, as assessed across various cognitive abilities (Barbosa et al., 2021). Notably, neurological disorders, including dementia, rank as the second leading cause of death and the primary cause of disability worldwide, with projections indicating that they will contribute to more than half of the economic burden of disability by 2050 (Feigin et al., 2020).

Situations that may impair cognitive performance in aged adults are primarily related, but not limited, to conditions directly affecting cognition, such as dementia (Jack et al., 2018), Mild Cognitive Impairment (MCI, Petersen et al., 2018), stroke (Huang et al., 2023), traumatic brain injuries (Torregrossa et al., 2023), or instances where treatment for an illness might influence cognitive abilities (e.g., chemotherapy, Cheung et al., 2012). The measurement of cognitive performance is typically conducted through a neuropsychological assessment, which usually involves different levels of investigation, from screening or case finding (first level) to diagnostic and differential evaluation (second level).

An approach that can be used to distinguish pathological from non-pathological changes in cognitive functions is based on the normal distribution of cognitive performance in a neurologically healthy sample (without evident signs of ongoing pathology) (Capitani and Laiacona, 2017). Based on this approach, an individual’s performance is compared to that of a healthy sample with the same characteristics that could influence performance, like age and education (Perry et al., 2017).

Brief screening measures of general cognitive status (e.g., Mini-Mental State Examination, MMSE, Folstein et al., 1975; Montreal Cognitive Assessment, MoCA, Nasreddine et al., 2005) have been developed in response to the need for early detection of cognitive deterioration. Such screening tests are expected to be relatively quick, easy to administer, score, and interpret, and could be carried out by professionals other than neuropsychologists, such as general practitioners, nurses, or trained staff. For example, regular cognitive screening is a recommended measure for the prevention of Alzheimer’s Disease for individuals over 50 years old (Alzheimer’s Association, 2019). However, despite the crucial role general practitioners play in detecting early signs of cognitive decline in older adults, only 16% of those in need receive regular cognitive function screenings (Alzheimer’s Association, 2019). The limited use of cognitive screening in these contexts is due to several factors: the nature of primary care settings (such as unsuitable office environments and limited time for visits), physicians’ specific competencies (including a lack of training in neuropsychological assessment, scoring, and interpretation), and contingent issues like the need for social distancing due to infectious risks, such as those associated with COVID-19 (Staffaroni et al., 2020).

Several systematic reviews have reported a remarkable increase in new technology-based cognitive assessment tools worldwide (Chan et al., 2021; Giaquinto et al., 2022; Pieri et al., 2023; Thabtah et al., 2020; Tsoy et al., 2021). Technological advancements could present an opportunity for overcoming these issues through self-administration, self-scoring, and reduced time involvement for professionals (Isaacson and Saif, 2020). Thus, their use in primary care seems appropriate (Sabbagh et al., 2020). Although some studies show that digital tools outperform traditional paper-and-pencil methods in distinguishing MCI and dementia from controls, most digital tools lack clinical, multilingual, and cross-cultural validation studies. These tools often have small sample sizes, are typically administered to highly educated individuals, and rarely fully leverage the advantages of digital formats over paper-and-pencil methods, such as the precise measurement of test completion times (Giaquinto et al., 2022). Indeed, some of these tools include tasks with time limits, but all of them can record the time taken to finish (response time), which reflects the duration of relevant cognitive operations, along with the time required for selecting and executing the response. The speed score may be a very important measure for detecting age-related variation in behavior in healthy aging and preclinical phases in dementing disorders (for a meta-analysis on AD disorder see Bäckman et al., 2005). Numerous studies suggest that normal aging gradually decreases processing speed, starting from early middle age (e.g., Zimprich and Mascherek, 2010). Processing speed declines as people grow older with whichever type of processing speed task is used: psychometric (e.g., Hedden and Gabrieli, 2004), cognitive-experimental (e.g., Der and Deary, 2006), or psychophysical (e.g., Gregory et al., 2008). It is important to note that the psychometric approach notably includes both cognitive tests with time limits (number of correct responses within predetermined time limits, e.g., the Attentive Matrices or the Wechsler Digit Symbol-Coding and Symbol Search), as well as tests that assess processing speed by measuring the time to complete tasks (e.g., the Trail Making Test -A). Both types of cognitive tests have been applied in clinical settings to assess processing speed in both normal and neurologically impaired populations, alongside other cognitive functions.

Therefore, considering the usefulness of easily administered and applied screening tools that are self-administered and have automatic corrections, along with the importance of having not only accuracy measures but also response time measures, digital tools undoubtedly meet these needs. Among at least 30 touchscreen tools available in 22 countries, only a few of them are available in multiple languages and culturally validated in different populations, and none of them has yet been validated in Italian (Giaquinto et al., 2022).

Recently, the Self-Administered Tasks Uncovering Risk of Neurodegeneration (SATURN) (Bissig et al., 2020) has been developed and validated in clinical settings in the United States. This freely accessible electronic screening tool proved comparable to the MoCA in identifying cognitive impairment. SATURN was originally introduced to assess the risk of cognitive symptoms that may indicate a potential neurodegenerative disorder. In the original study (Bissig et al., 2020), it was tested on patients from a dementia clinic who had Alzheimer’s Disease, other forms of dementia, or other neurological conditions such as Multiple Sclerosis, Essential Tremor, or Parkinson’s disease. Like the MMSE and MoCA, it can be useful in various conditions where cognitive symptoms emerge in domains such as attention, memory, orientation, calculation, visuo-constructional abilities, and executive functions, which are characteristic of pathological cognitive aging. SATURN relies solely on visual stimuli, requiring some reading ability, thus overcoming potential language barriers between examiners and patients, or prevalent hearing impairments in the target population. It also reduces hardware and software demand for remote use, such as eliminating the need for speaker volume calibration and multimodal stimuli for time synchronization. SATURN is self-administered, automatically scored, and open source for professionals and researchers, and it was comparable to MoCA, for example, in detecting dementia from controls. Its sensitivity and specificity in the USA population were 0.92 and 0.88, respectively (Bissig et al., 2020). Thanks to its open-source nature, which facilitates its dissemination, it has already been adapted into other languages (e.g., Korean, Vietnamese, simplified Chinese, Italian, and Greek), although clinical or normative validation data for the tool outside of the USA have not yet been published.

This study aims to establish normative data for the Italian version of SATURN, using a sample of individuals aged 50–80 years, stratified by age, sex, and education. Conducting a normative study on SATURN in the Italian context would enhance knowledge of the tool’s psychometric characteristics, facilitate its multilingual and cross-cultural dissemination, provide insights into its performance among individuals with lower education levels than those in the original sample, and offer an estimate of administration times that were not included in the original study. Several innovative aspects distinguish it from the validation of the original version. In the Italian version, we analyzed both time on task and accuracy for each task and cognitive subdomain (represented by an average of tasks exploring each specific cognitive subdomain). The total time on tasks for the entire test was also computed. A standardization methodology outlined by Capitani and Laiacona (2017), which is the accepted standard for validating neuropsychological tests in Italy, was applied to the accuracy and time on task data. Another noteworthy feature is the inclusion of a preliminary assessment of visual acuity and color perception, aimed at identifying potential visual impairments that could impact task performance or render the test inapplicable. Additionally, to facilitate users, an automatic scoring function has been implemented. After completing the digital test, users can remotely download a report containing raw, adjusted, and equivalent scores for each variable. A local version of the tool is accessible via PsychoPy and can also be remotely administered through Pavlovia. It is openly shared through a specified link (see Methods) and allows for future software updates to be downloaded.

## 2 Methods

### 2.1 Subjects

A total of 330 subjects living in the region of Puglia (Italy) were recruited. A balanced sampling method based on age, education, and sex was used. Each administrator was assigned a specific age and education group, with the request to carry out a balanced number of assessments for each sex. Two recruitment strategies were employed in two different phases of the research. The recruitment strategy was initially conducted directly, using flyers posted in pharmacies, doctors’ offices, and at events promoting cognitive prevention. Subsequently, a snowball sampling method was employed to specifically target and complete the necessary strata of the population. Inclusion criteria were: (i) age between 50 and 80 years; (ii) absence of self-reported diagnosis of neurologic diseases (e.g., MCI, any kind of dementia, stroke, traumatic brain injuries); (iii) MoCA equivalent score ≥1. Participants with other well-known risk factors for dementia (e.g., alcohol abuse, hypertension, diabetes, history of depression, etc.), as identified by Livingston et al. (2020), but without a diagnosis of dementia, were included to ensure the representation of the general population. We excluded from analysis: (i) participants (*n* = 3) who achieved an age- and education-adjusted MoCA equivalent score equal to 0 [according to the normative Italian data by Santangelo et al., 2015], to reduce probability of enrolling individuals with possible diffuse cognitive impairment, as it has been done in previous normative studies on Italian neuropsychological tests (e.g., Conti et al., 2015; Santangelo et al., 2015); (ii) participants (*n* = 2) whose total completion time on SATURN was higher than three standard deviations from the sample mean, as outliers due to unintentional interruptions during administration; (iii) participants (*n* = 2) with 0 years of formal education, as extremely rare cases that cannot be considered in the age-education classification used in this study.

All participants took part in the study on a voluntary basis after having provided their written informed consent and without receiving any reward. This enrolment procedure resulted in a sample of 323 neurologically healthy Italian subjects (180 females and 143 males), representing all levels of formal education based on the three-level coding system from the International Standard Classification of Education (ISCED) of the United Nations Educational, Scientific and Cultural Organization (UNESCO), as suggested by Boccardi et al. (2022): compulsory (primary and secondary) education; upper-secondary education; and post-secondary education. In Italy, the first level corresponds to less than 9 years of education, the second level from 9 to 13 years, and the third level more than 13 years of education. The mean age of the whole sample was 61.33 ± 8.75 years, mean formal education was 11.32 ± 4.59 years. The distribution of the sample for age and education is reported in Table 1.

**Table 1:** Demographic distribution of the sample are reported in terms of age (50–59, 60–69, 70–80 years old), years of education (<9, 9–13, >13 years), and sex (male, female).

		Age—years			
Sex	Years of education	50–59	60–69	70–80	Total
Male	< 9	27	12	21	60
	9–13	28	14	7	49
	>13	16	15	3	34
	Total	71	41	31	143
Female	<9	36	14	28	78
	9–13	34	21	5	60
	>13	21	16	5	42
	Total	91	51	38	180
Total	<9	63	26	49	138
	9–13	62	35	12	109
	>13	37	31	8	76
	Total	162	92	69	323

### 2.2 Procedure

Participants were briefed about the study and signed an informed consent form. Data collection took place in a quiet environment where the participant was alone with the experimenter conducting the evaluation. The research protocol consisted of paper-and-pencil tests and digital tests administered via a laptop with mouse input (hardware) using PsychoPy software version 2022.2.2. The paper-and-pencil protocol was administered by the experimenter, while the digital one was self-administered. Only in cases where participants were unable to use the computer, the experimenter could offer limited assistance with entering responses, without influencing reading, comprehension of the text, or completion of the tests in any way. The protocol was the same and in the same order for all participants. The administration occurred in a single session and by a single experimenter. The digital and paper-and-pencil protocols were administered by psychologists who were adequately trained. The administration lasted approximately 30 min. Data collection took place from April 2023 to December 2023. The study received approval from the National Ethics Committee for experiments of public research institutions (NEC) and other national public entities (CEN) during the session held on April 4, 2023, Project Code: PNRR-MAD-2022-12376781.

### 2.3 Measures

#### 2.3.1 Paper-and-pencil protocol

The Mini-Mental State Examination (MMSE, Folstein et al., 1975), administered orally, is a widely used tool assessing cognitive functions in individuals with suspected cognitive impairment. It evaluates various cognitive areas, including temporal and spatial orientation, short-term memory, attention, and calculation ability. It comprises a series of questions and tasks, with a maximum score of 30 points, where lower scores indicate greater cognitive impairment. In the updated normative study that enrolled people from the South of Italy (Carpinelli Mazzi et al., 2020), the multiple linear regression showed a significant effect of age and education on the raw score, but not for sex, and the cut-off score fixed to 24.9/30 led to detect 44 out of 47 Alzheimer’s Disease patients.

The Montreal Cognitive Assessment (MoCA, Nasreddine et al., 2005) is a cognitive screening tool developed to detect MCI. It assesses multiple cognitive domains, including memory, language, executive functions, visuospatial abilities, calculation, abstraction, attention, concentration, and orientation. Like the MMSE, it consists of various questions and tasks with a maximum score of 30 points, where lower scores indicate cognitive issues. In the Italian normative study considered here (Santangelo et al., 2015), the multiple linear regression showed a significant influence of age and education on the raw score, but not for sex. The inferential cut-off score was fixed at 15.5. The correlation between MoCA and MMSE-adjusted scores was statistically significant and medium positive (*r* = 0.43; *p* < 0.001).

#### 2.3.2 Digital protocol

The SATURN (Bissig et al., 2020) is a self-administered digital test designed to assess global cognitive functioning and detect cognitive decline. Originally consisting of 20 brief cognitive tasks, it assessed a range of cognitive functions such as memory, attention, temporal orientation, visuo-constructional abilities, math (calculation), and executive functions in a time limit of 15 min (the program ends when one completes any tasks started within 15 min). In the American validation study, the total accuracy and the reading time (based on the duration participants spent reading verbal instructions for specific tasks, excluding any figures), were computed. Recently, the SATURN was adapted to test its remote, completely unsupervised delivery and usability in a relatively large group of healthy older adults recruited online (Tagliabue et al., 2023). The Italian version of the Saturn test was translated and adapted from its original English counterpart. Saturn primarily relies on visual tasks (see Tagliabue et al., 2023). The components requiring translation, apart from the instructions, were the attention, incidental memory, and recall items. For these items, direct translation of the English words was not employed. Instead, word selection was based on the Lexvar database (Barca et al., 2001; Barca et al., 2002) using the following procedure. First, the word database was ranked by familiarity (i.e., the extent to which a word is encountered in daily life, provided within the Lexvar) and frequency (Bertinetto et al., 2005). Only words of 4, 5, or 6 letters in length were considered. Subsequently, a composite score was calculated by averaging the z-transformed scores for familiarity and frequency for each word. Words falling within the third and fourth quartiles of this composite score distribution were then randomly selected to be used in the SATURN items. Efforts were made to balance word length (4, 5, and 6 letters) within tasks. The instructions were freely translated from English into Italian, with an emphasis on ensuring clarity and comprehensibility for the target population. The adapted version of SATURN was pilot-tested in a laboratory setting with older adult participants, who completed the SATURN test independently on a tablet and provided verbal feedback on the translation and usability. The Italian version was implemented using PsychoPy® (v2022.2.2) (Peirce et al., 2019) and can be translated into JavaScript, and hosted on Pavlovia^1^ for remote administration. PsychoPy and Pavlovia are products of Open Science Tools Ltd^2^, available for free or with a small fee, respectively. For consistent online unsupervised administration, the Italian SATURN differs from the original American version in certain aspects: the introductory sentence (“Click on the square to proceed” instead of “Close your eyes”), the instruction for the incidental memory task (“What shape had been selected?” instead of “What phrase was read at the start?”), and the removal of the spatial orientation question, which could not be automatically scored. The final Italian version comprises 19 tasks with a maximum total score of 29. Description of each task is reported in Table 2. In the present validation study, given the program’s valuable capability to record the time (expressed in seconds) spent on each task (hereafter referred to as ‘time on task’), we decided to analyze both accuracy and time on task for each cognitive subdomain. Each subdomain is represented by an average of accuracies and times on tasks exploring that specific subdomain. Additionally, we examined the SATURN total accuracy and total time spent on tasks by summing the accuracy scores and the time spent on each of the 19 tasks that constitute the test. Similarly to the American version, a reading time determined by the duration participants spent reading verbal instructions for specific tasks (excluding any figures) was also calculated. Moreover, in the Italian PsychoPy® version used in this study the tasks’ administration is preceded by a measure of visual acuity and color perception aimed at examining the subsistence of the examination conditions. SATURN demonstrated excellent feasibility features such as cross-platform portability, satisfactory user experience, and clarity of instructions [to delve deeper into the characteristics of the remote online adaptation, please refer to Tagliabue et al., 2023]. An updated version of Italian SATURN is publicly available at: https://osf.io/cdmt3/?view_only=44c8bc4977274e02969a2d7b80362caf.

**Table 2:** Here are reported the subdomains assessed with the SATURN, the tasks used to assess each domains, and the instruction/description of the task.

Subdomain	Task	Instruction/Description
Attention	G – word	“Pick the word that starts with G” in a list of 10 words
	Fruit words	“Pick the fruits word” in a list of 4 words
	Number	“Type in the number 1239” using a numeric keypad
Incidental memory	Shape	“Earlier, what shape did I ask you to click on this screen?”
	Word	“Remember your first task when you got this tablet? You had to pick a single word starting with a certain letter. Pick the same word, again”
	Number	“When you first saw this number pad, you had to enter a specific four-digit number. Enter the same number, again”
Orientation	Month	“What month is it?”
	Year	“What year is it?”
	Day	“What day of the week is it, today?”
Recall	Recall of five words	Recognize 5 words previously learnt in a list of 100 words.
Math	Sum	“You go to the store with exactly $100. You buy a dozen apples for $7 and a tricycle for $60. How much did you spend?”
	Difference	“After the purchase, how much do you have left?”
Visuo-constructional abilities	Shape	“Next, you will see some drawings. Pick the small drawings that make up the big drawing.” A circle and a penthagon are showed as shapes.
	Face	Same instruction, with faces as symbols.
	Line	Same instruction, with lines as symbols.
	Cube	Same instruction, with cubes as symbols.
Executive functions	STROOP	“Next, you will see some words. Choose the color used to write each word.” This is a brief version of the Stroop task.
	TMT numbers	“Connect the dots. Start at number 1, and go in order. Keep going until you reach the end.”
	TMT numbers and letters	“Connect the dots. Alternate numbers and letters. Start at 1, connect to the first letter, a. Then, connect a to 2, and 2 to the next letter. Keep going until you reach the end.” This is a Trial Making Test task.

### 2.4 Statistical analysis

Statistical analysis followed the methodology outlined by Capitani and Laiacona (2017), which is the accepted standard for validating neuropsychological tests in Italy. Multiple regression analyses were conducted to determine the impact of demographic variables (such as sex, age, and education) on participants’ SATURN total accuracy and total time spent on tasks, as well as accuracy and time spent on tasks in each subdomain, and on the reading time for task instructions. Sex, age, and education were included in a multiple linear regression analysis to account for their potential overlapping effects. The results of these analyses were used to generate correction factors for each subject in the sample, which were applied to adjust raw scores. Adjusted scores were then ranked and categorized into a five-point interval scale (Equivalent Score, ES), ranging from 0 to 4, with specific criteria for each category. To identify normal and abnormal scores, non-parametric procedures (Capitani and Laiacona, 2017) were employed to calculate outer and inner tolerance limits, based on the distribution of scores within the normal population. Confidence intervals were set at 95%. Correction formulas and grids were provided to adjust raw scores based on demographic variables, such as age and education level. Correlations were calculated using Person’s *r*, and significance was set at *p* < 0.05. Analysis was run using JASP (JASP Team, 2024).

## 3 Results

SATURN mean total accuracy was 24.02 ± 3.25, ranging from 14 to 29, while mean total time on tasks was 403.09 ± 194.50 s (6.72 ± 3.24 min), ranging from 76.82 to 1172.15 s (i.e., 1.28–19.54 min). Table 3 reports descriptive statistics for demographic characteristics (age, education), raw and adjusted scores of the MMSE and MoCA, and SATURN total and subdomain accuracies and times on tasks. Approximately, 70–80 years old participants performed worse in SATURN total accuracy than 50–59 years old, and the less educated subjects had the lowest total accuracy. The distribution of SATURN mean total accuracy as a function of age and education is reported in Table 4. The mean distribution of accuracy for each subdomain, as well as the total and subdomain times on tasks, is reported in the Supplementary Table S1.

**Table 3:** Descriptive statistics.

	Median	Mean	St. Dev.	Min.	Max.
Age	59.00	61.33	8.75	50.00	80.00
Education (years of)	11.00	11.32	4.59	2.00	30.00
MMSE raws	29.00	28.27	2.08	19.00	30.00
MMSE adjusted	28.83	28.33	2.01	18.56	30.00
MoCA raws	25.00	24.53	3.33	13.00	30.00
MoCA adjusted	25.40	25.06	3.34	16.22	30.00
SATURN accuracy					
SATURN total accuracy	25.00	24.02	3.25	14.00	29.00
Attention mean score	2.00	1.95	0.20	<0.01	2.00
Incidental memory mean score	1.00	0.87	0.19	0.33	1.00
Orientation mean score	1.00	0.84	0.18	0.33	1.00
Recall memory mean score	3.00	3.22	1.20	<0.01	5.00
Math mean score	1.50	1.28	0.45	<0.01	1.50
Visuo-constructional mean score	0.75	0.78	0.24	<0.01	1.00
Executive functions mean score	1.67	1.38	0.38	0.33	1.67
SATURN times on task (seconds)					
SATURN total time on tasks	385.80	403.09	194.50	76.82	1172.15
Attention time mean score	9.20	10.11	5.89	2.19	44.73
Incidental memory time mean score	10.99	13.81	10.52	2.05	85.12
Orientation time mean score	6.38	7.37	4.21	2.07	48.93
Recall memory time mean score	79.97	94.42	65.19	11.69	394.08
Math time mean score	18.42	22.28	19.65	2.22	210.97
Visuo-constructional time mean score	18.12	19.98	14.60	1.66	101.09
Executive functions time mean score	27.29	30.11	13.53	6.96	87.94
Reading time mean score	13.23	12.99	7.35	1.62	33.83

Median, mean, Standard Deviation (St. Dev.), Minimum (Min.) and Maximum (Max.) are reported for demographics characteristics (age, education), for raw and adjusted scores of Mini Mental State Examination (MMSE), and Montreal Cognitive Assessment (MoCA). For SATURN, total accuracy and total times on task are presented. For each sub-domains, mean score is presented. The time spent from the subjects to complete each task is calculated by the sum of seconds spent for each task (SATURN total time on tasks). For each sub-domains, time on task mean score is presented. Reading time is calculated as a mean of times spent to read tasks’ verbal instructions (Reading time mean score).

**Table 4:** SATURN total mean scores (±SD) as a function of age and education.

SATURN accuracy, Mean (standard deviation)				
	Age—years			
Years of education	50–59	60–69	70–80	Total
<9	24.11(2.89)	21.88(3.79)	23.29(3.70)	23.40(3.44)
9–13	24.76(3.14)	23.63(2.85)	25.08(3.00)	24.43(3.06)
>13	25.54(2.56)	24.03(2.76)	22.00(4.11)	24.53(3.01)
Total	24.69(2.95)	23.27(3.21)	23.45(3.68)	24.02(3.25)

A series of multiple linear regression models revealed the influence (or lack thereof) of sociodemographic variables (sex, age, education) on total and subdomain accuracies and times on task. A significant effect of age and education emerged for SATURN total accuracy, while sex significantly influences the total time on tasks. Subdomain accuracies and times are influenced differently by sociodemographic characteristics. A significant effect of sex emerged for math accuracy and time on tasks in the subdomains of math, visuo-constructional abilities, and executive functions. A significant effect of age emerged for incidental memory accuracy, recall memory accuracy, visuo-constructional abilities accuracy, executive functions accuracy and executive functions time. A significant effect of education emerged for orientation accuracy, recall memory accuracy, visuo-constructional abilities accuracy, attention time and incidental memory time (Table 5). We derived the formulas from multiple linear regression models to calculate the adjusted scores for SATURN total and subdomain accuracies and times on tasks, based on age, sex, and education (Table 6).

**Table 5:** Multiple linear regression and beta unstandardized coefficients for the SATURN total accuracy, total time on tasks, and accuracy and time on tasks on each cognitive subdomain.

Cognitive subdomains	*R*	*R^2^*	*F*	*P*	Beta (unstandardized coefficients)		
					Sex	Age	Education
SATURN total accuracy	0.256	0.065	7.436	<0.001	ns	−0.062	0.106
SATURN total time on tasks	0.182	0.033	3.655	0.013	68.421	Ns	ns
SATURN subdomain (accuracy)							
Attention	0.104	0.011	1.164	ns	ns	ns	ns
Incidental memory	0.169	0.029	3.123	0.026	ns	−0.003	ns
Orientation	0.252	0.063	7.189	<0.001	ns	ns	−0.008
Recall memory	0.301	0.091	10.608	<0.001	ns	−0.028	0.046
Math	0.157	0.025	2.703	0.046	−0.105	ns	ns
Visuo-constructional abilities	0.310	0.096	11.286	<0.001	ns	−0.003	0.013
Executive functions	0.127	0.016	1.755	ns	ns	−0.005	ns
SATURN Subdomain (time on tasks)							
Attention	0.178	0.032	3.486	0.016	ns	ns	−0.151
Incidental memory	0.165	0.027	2.964	0.032	ns	ns	ns
Orientation	0.159	0.025	2.758	0.042	ns	ns	−0.149
Recall memory	0.079	0.006	0.664	ns	ns	ns	ns
Math time	0.221	0.049	5.455	0.001	7.738	ns	ns
Visuospatial	0.229	0.052	5.879	<0.001	6.496	ns	ns
Executive functions	0.183	0.034	3.704	0.012	3.069	0.216	ns
Reading time	0.125	0.016	1.685	0.170	ns	ns	ns

**Table 6:** Correction formulas for SATURN total and subdomains accuracies and time on tasks, according to sex, age and education.

	**Adjustement formula**
SATURN total accuracy	Raw + [(−0.106 x (educ – 11.316)) + (0.062 x (age – 61.334))]
SATURN total time in tasks	If female: Raw – 68.42
SATURN subdomain (accuracy)	
Attention	No adjustment
Incidental memory	Raw + [(0.003 × (age – 61.334)]
Orientation	Raw + [(0.008 × (educ – 11.316)]
Recall memory	Raw + [(−0.046 × (educ – 11.316)) + (0.028 × (age – 61.334))]
Math	If female: Raw +0.11
Visuo-constructional abilities	Raw + [(−0.013 × (educ – 11.316)) + (0.003 × (age – 61.334))]
Executive function	Raw + [(0.005 × (age – 61.334))]
SATURN subdomain (time on tasks)	
Attention	Raw + [(0.151 × (educ – 11.316))]
Incidental memory	No adjustment
Orientation	Raw + [(0.149 × (educ – 11.316))]
Recall memory	No adjustment
Math	If female: Raw −7.74
Visuo-constructional abilities	If female: Raw −6.50
Executive function	Raw + (−0.216 × (age – 61.334))] + *(if female)*(−3.07)
Reading speed	No adjustment

Considering a sample of 323 subjects, outer and inner non-parametric tolerance limits are defined by values corresponding to the 10th and 24th worst observation. The outer limit for adjusted SATURN total accuracy corresponds to 16.56, while the inner to 18.57. The outer limit for adjusted SATURN total time on tasks corresponds to 832.83 s, while the inner to 706.56 s. The outer tolerance limit corresponds to the cut-off point, and scores falling beneath it (or above, if we consider times) may be seen as abnormal since it also encompasses the value of the 10th lowest observation (Capitani and Laiacona, 2017). Adjusted score higher (or lower, if we consider times) than the inner tolerance limit indicates a performance that we are 95% confident could come from the highest 95% of the distribution of the healthy population. Scores between outer and inner limits correspond to the 4.33% of our sample and are associated with borderline performance. Table 7 displays the inner and outer non-parametric tolerance limits, ES, the number of individuals within each ES (density), and the total frequency of individuals from 0 to 1, 2, 3, and 4 for the total and each subdomain score and time. For attention accuracy, we reported only Inner and Outer tolerance limits because of a ceiling effect: most individuals reached the Inner tolerance score, making not possible to distinguish different equivalent scores.

**Table 7:** Equivalent scores (ES) for the SATURN total and subdomain accuracies and time on tasks.

Equivalent scores	Interval	Cumulative frequency	Density
SATURN total accuracy			
Outer npTL	16.56	10	10
Inner npTL	18.57	24	313
0	≤16.56 (cut-off)	10	10
1	16.56–19.80	36	26
2	19.80–22.40	88	52
3	22.40–24.68	161	73
4	>24.68	323	162
SATURN total time on tasks			
Outer npTL	832.83	10	10
Inner npTL	706.56	24	313
0	≥832.83	10	10
1	832.83–662.92	36	26
2	662.92–539.37	88	52
3	539.37–434.49	161	73
4	<434.49	323	162
SATURN Subdomain (accuracy)			
Attention			
Outer npTL	1.33	10	10
Inner npTL	2.00	24	313
Incidental memory			
Outer npTL	0.36	10	10
Inner npTL	0.63	24	313
0	≤0.36	10	10
1	0.36 – 0.65	36	26
2	0.65–0.69	88	52
3	0.69 – 0.97	161	73
4	>0.97	323	162
Orientation			
Outer npTL	0.62	10	10
Inner npTL	0.64	24	313
0	≤0.62	10	10
1	0.62–0.64	36	26
2	0.64–0.68	88	52
3	0.68–0.95	161	73
4	>0.95	323	162
Recall memory			
Outer npTL	0.99	10	10
Inner npTL	1.39	24	313
0	≤0.99	10	10
1	0.99–1.70	36	26
2	1.70–2.60	88	52
3	2.60–3.19	161	73
4	>3.19	323	162
Math			
Outer npTL	0.11	10	10
Inner npTL	0.50	24	313
0	≤0.11	10	10
1	0.11–0.61	36	26
2	0.61–1.50	88	52
3	1.50–1.50	161	73
4	>1.50	323	162
Visuo-constructional abilities			
Outer npTL	0.30	10	10
Inner npTL	0.44	24	313
0	≤0.30	10	10
1	0.30 – 0.48	36	26
2	0.48 – 0.64	88	52
3	0.64 – 0.80	161	73
4	> 0.80	323	162
Executive functions			
Outer npTL	0.37	10	10
Inner npTL	0.67	24	313
0	0.37	10	10
1	0.73	36	26
2	1.29	88	52
3	1.61	161	73
4	>1.61	323	162
SATURN Subdomain (time on tasks)			
Attention			
Outer npTL	26.51	10	10
Inner npTL	18.48	24	313
0	≥26.51	10	10
1	26.51–16.42	36	26
2	16.42–11.62	88	52
3	11.62–9.66	161	73
4	<9.66	323	162
Incidental memory			
Outer npTL	35.67	10	10
Inner npTL	28.57	24	313
0	≥35.67	10	10
1	35.67–24.44	36	26
2	24.44–16.30	88	52
3	16.30–11.02	161	73
4	<11.02	323	162
Orientation			
Outer npTL	16.25	10	10
Inner npTL	12.97	24	313
0	≥16.25	10	10
1	16.25–11.36	36	26
2	11.36–8.27	88	52
3	8.27–6.58	161	73
4	<6.58	323	162
Recall memory			
Outer npTL	244.64	10	10
Inner npTL	189.07	24	313
0	≥244.64	10	10
1	244.64–174.33	36	26
2	174.33–128.91	88	52
3	128.91–81.40	161	73
4	<81.40	323	162
Math			
Outer npTL	65.00	10	10
Inner npTL	42.52	24	313
0	≥65.00	10	10
1	65.00–36.44	36	26
2	36.44–21.95	88	52
3	21.95–14.79	161	73
4	<14.79	323	162
Visuo-constructional abilities			
Outer npTL	47.73	10	10
Inner npTL	38.53	24	313
0	≥47.73	10	10
1	47.73–33.04	36	26
2	33.04–22.00	88	52
3	22.00–14.49	161	73
4	<14.49	323	162
Executive functions			
Outer npTL	61.89	10	10
Inner npTL	46.28	24	313
0	≥61.89	10	10
1	61.89–43.78	36	26
2	43.78–34.93	88	52
3	34.93–25.89	161	73
4	<25.89	323	162
Reading time			
Outer npTL	27.59	10	10
Inner npTL	24.29	24	313
0	≥27.59	10	10
1	27.59–21.97	36	26
2	21.97–17.72	88	52
3	17.72–13.24	161	73
4	<13.24	323	162

Times are expressed in seconds.

A correction grid was devised to accommodate the raw scores of tested individuals based on demographic factors. This grid was tailored for common combinations of age (grouped in 10-year increments) and educational attainment. In cases where the correction grid does not cover specific demographic profiles, regression equations can be employed to estimate adjusted scores (Table 8).

**Table 8:** Correction grid for SATURN total and subdomain accuracies and time on tasks, according to age and education.

Education – years	Age - years		
	50–59	60–69	70–80
SATURN total accuracy			
<9	−0.086	+0.584	+1.413
9–13	−0.539	+0.155	+0.703
>13	−1.109	−0.591	−0.007
SATURN total time on tasks			
<9	−2.27	−98.40	−220.64
9–13	+79.52	−20.67	−92.76
>13	+182.09	+111.83	+26.58
SATURN Subdomain (accuracy)			
Attention			
<9	/	/	/
9–13	/	/	/
>13	/	/	/
Incidental memory			
<9	−0.022	+0.008	+0.041
9–13	−0.022	+0.009	+0.042
>13	−0.021	+0.008	+0.032
Orientation			
<9	−0.028	−0.032	−0.042
9–13	+0.007	+0.002	+0.013
>13	+0.051	+0.019	+0.050
Recall memory			
<9	−0.045	+0.256	+0.628
9–13	−0.242	+0.070	+0.321
>13	+0.103	+0.125	+0.061
Math			
<9	+0.060	+0.057	+0.060
9–13	+0.058	+0.063	+0.044
>13	+0.060	+0.054	+0.066
Visuo-constructional abilities			
<9	+0.024	+0.059	+0.110
9–13	−0.033	+0.005	+0.021
>13	−0.104	−0.084	−0.050
Executive functions			
<9	−0.037	+0.013	+0.069
9–13	−0.036	+0.015	+0.070
>13	−0.035	+0.013	+0.053
SATURN Subdomain (time on tasks)			
Attention			
<9	−0.54	−0.60	−0.80
9–13	+0.13	+0.04	+0.24
>13	+0.96	+1.06	+0.95
Incidental memory			
<9	/	/	/
9–13	/	/	/
>13	/	/	/
Orientation			
<9	−0.53	−0.59	−0.79
9–13	+0.13	+0.04	+0.24
>13	+0.94	+1.05	+0.94
Recall memory			
< 9	/	/	/
9–13	/	/	/
>13	/	/	/
Math			
<9	−4.42	−4.17	−4.42
9–13	−4.24	−4.64	−3.22
>13	−4.39	−3.99	+4.01
Visuo-constructional abilities			
<9	−3.71	−3.50	−3.72
9–13	−3.56	−3.90	−2.71
>13	−3.69	−3.35	−4.06
Executive functions			
<9	−0.14	−2.22	−4.73
9–13	−0.12	−2.49	−4.32
>13	−0.22	−2.13	−4.22
Reading speed			
<9	/	/	/
9–13	/	/	/
>13	/	/	/

Examination of the correlation coefficient between the adjusted SATURN total accuracy and MMSE adjusted scores indicated a statistically significant small positive correlation (*r* = 0.19, *p* < 0.001), the correlation between the adjusted SATURN total accuracy and MoCA adjusted scores was statistically significant, positive, and more consistent (*r* = 0.30, *p* < 0.001), like that between MoCA and MMSE (*r* = 0.31, *p* < 0.001).

A total of 86 (26.6%) individuals received assistance during the completion of SATURN because of self-reported absence of experience in using computers. They were older [Age help, yes: 67.98 ± 9.09; no: 58.92 ± 7.26, *t*(321) = −9.234, *p* < 0.001] and less educated [Education help, yes: 8.41 ± 3.30; no: 12.37 ± 4.56, *t*(321) = 7.420, *p* < 0.001] than people who did not required assistance, with no significant sex difference (*X*^2^ = 0.074; *p* = 0.785). To test the influence of receiving assistance on SATURN total accuracy, total time on tasks, and reading speed, we performed a series of multiple linear regression models considering sex, age, education, and assistance received as factors. Results confirmed the influencing factors reported in Table 5 and receiving assistance did not affect any outcome (Supplementary Table S2).

## 4 Discussion

This research is the pioneering effort to provide normative data stratified by age, education, and sex for the Italian version of SATURN, the first open-source digital tool for the initial assessment of cognitive functions to be introduced in the Italian context. The distinctive features of the tool, such as self-administration, automatic correction, and instant report generation, offer fundamental advantages that traditional paper-and-pencil methods cannot provide and could facilitate the application in primary care setting (Staffaroni et al., 2020). The availability of the same tool in different languages facilitates both the development of research in different countries and the clinical use of the tool with diverse populations (Canevelli et al., 2022; Giaquinto et al., 2022).

Compared to the original study in the USA (Bissig et al., 2020) and the previous online one (Tagliabue et al., 2023), the average education level of the sample was lower (USA: 16.3 ± 2.5; Tagliabue et al., 2023: 15.7 ± 3.1; this study: 11.3 ± 4.6), but in line with the demographic characteristics of Southern Italy (Caffò et al., 2016; Giaquinto et al., 2023). Probably for this reason, the average raw score is lower in this study (USA: 27.1 ± 2.6 out of 30; Tagliabue et al., 2023: 27.0 ± 1.7 out of 29; this study: 24.0 ± 3.3 out of 29). The current study provides normative data for SATURN along with cutoff values for detecting potential cognitive decline. The cutoff value (16.56 out of 29) derived in this study is much lower than the one reported by Bissig et al. (2020) (21.00 out of 30). However, the influence of age and education on total raw accuracy is in line with correlations reported in Tagliabue et al. (2023), and with multiple regressions reported in MMSE (Carpinelli Mazzi et al., 2020) and MoCA (Santangelo et al., 2015) Italian normative studies. The Italian version of SATURN significantly correlates with MMSE and MoCA (positive correlations), ranging from small to moderate, respectively. The correlation between SATURN and MoCA in the original study was significantly higher (*r* = 0.90; *p* < 0.001), but this was consistent with a sample composed of both patients and controls. In fact, the correlation between SATURN, MoCA, and MMSE reflects the moderate positive correlation between MoCA and MMSE, after adjusting for age and education, observed in previous studies on normative Italian samples (e.g., Santangelo et al., 2015: *r* = 0.43, *p* < 0.001; Conti et al., 2015: *r* = 0.32, *p* < 0.001). These results demonstrate the convergent validity of SATURN on normative samples. Further studies exploring the convergent validity of SATURN on clinical samples are needed. Although the small correlation is not ideal for tests that aim to measure the same function, it remains unclear whether it is advantageous or not to overlap with the aforementioned screening tests that are known to have some severe limitations (Carnero-Pardo, 2014; Moafmashhadi and Koski, 2013). Moreover, the SATURN may tap more into cognitive functions barely touched by the MMSE, which is known to strongly rely on verbal abilities (e.g., attentional and executive functions). Finally, the fact that MMSE and MoCA are bounded scores, with many participants reaching a ceiling performance in a healthy population, may deflate the correlation because of a restriction of range artifact. Nonetheless, the significant correlation among these tests is consistent with the idea that they offer an assessment of overall cognitive functioning through a multi-domain approach, although they evaluate different cognitive domains, with only partial overlap (Ciesielska et al., 2016; Tagliabue et al., 2023).

As it was done for the Italian normative validation of the MoCA (Santangelo et al., 2015), normative scores for all cognitive subdomains assessed by the SATURN were also calculated. This was done to obtain a more precise understanding of the cognitive domains that might influence an overall performance below the norm and to guide second-level evaluation more accurately. Age and years of education influence performance in visuo-constructional tasks in SATURN, as well as in MoCA; some differences emerged about executive functions, influenced by age and education in MoCA but only age in SATURN, and orientation, influenced by age and education in MoCA but only education in SATURN. Age also influences incidental and recall memory in SATURN, consistently with MoCA. Females performed worse than males in math (calculation), and sex results as an influent variable for this subdomain. The current results are consistent with previous findings on male vs. female differences across the lifespan in math abilities (Kaufman et al., 2009) although it is more frequently studied on young adult samples involving college students.

This study leverages the capabilities of digital tools to provide normative values for times on task, which are automatically calculated. These values include the total and subdomain times spent on tasks, as well as the time taken to read the verbal instructions. Measuring time on tasks can help assess the possible presence of cognitive slowing, accordingly to the processing speed hypothesis (Salthouse, 1996, 2000). Clinically, detecting individuals at risk for dementing disorders early on is essential for enhancing treatment effectiveness and guiding other clinical and social decisions (Liss et al., 2021). The utility of considering the temporal parameter in executing cognitive tasks for detecting patients with MCI or those at greater risk of developing dementia has been demonstrated in various contexts. Psychometric tests, such as the WAIS digit-symbol test [see the meta-analysis by Bäckman et al., 2005], and experimental tests measuring reaction times (e.g., Schmidt et al., 2020) have shown significant findings. Additionally, ecological studies, including measuring response times in online surveys (e.g., Schneider et al., 2023) or gait speed to detect motor decline (e.g., Hackett et al., 2018; Grande et al., 2019), have also provided valuable insights. Some digital tools represent an effort in this direction (Giaquinto et al., 2022). For example, CognICA (Kalafatis et al., 2021) is a 5-min, self-administered tool that assesses Information Processing Speed, a key indicator of cognitive impairment, using a language-independent task. An AI algorithm analyzes accuracy, response speed, and age to estimate the likelihood of impairment by comparing the patient’s performance to healthy and impaired individuals. This highlights the potential of digital tools and machine learning in advancing neuropsychological assessments (Battista et al., 2020). This study provides SATURN normative values for time on task and reading times, which could be tested as discriminant variables in distinguishing cognitive decline from controls, either alone or in combination with accuracy, based on age and education level. Future studies are needed to explore these possibilities.

### 4.1 Limitations

It is important to consider certain limitations when discussing the results. The type of sampling and recruitment methods used, while effective for obtaining a stratified sample, may be susceptible to selection and response bias from individuals who frequently visit pharmacies, doctors’ offices, and awareness events. Additionally, snowball sampling could lead to the creation of a homogeneous sample. Furthermore, residing in a single region of Southern Italy may render the results of this study not generalizable to a sample of residents in the Northern part of the country, as is the case, for example, with the normative sample of the MMSE (e.g., Carpinelli Mazzi et al., 2020). The inclusion criteria used do not allow us to definitively exclude the possibility that individuals with various types of cognitive deficits may be present in the normative sample. However, this is also due to the limitations of existing paper-and-pencil screening tools, which do not achieve perfect sensitivity and specificity, and we do not know to what extent these limitations are also present in the Italian version of SATURN. Future clinical validity studies will need to assess the discriminative capacity of SATURN. Results also showed that older and less educated individuals, without computer experience, more often required assistance in submitting answers during the completion of SATURN. Although this did not affect the final results, it could represent a limitation for the remote application of SATURN in certain populations. Finally, although we believe that completion times for the various SATURN tasks and for the overall SATURN test are clinically useful measures of processing speed, they may include some noise.

## 5 Conclusion

Future studies need to evaluate the sensitivity and specificity values of the Italian version of the SATURN in identifying cases of pathological cognitive decline. To test the clinical application of the tool in primary care settings, a feasibility study is required to test its application in clinical practice. Preliminary results from this study offer encouraging prospects, as the self-administered tool showed no significant influence when minimal assistance was required for inputting questions, especially with no prior computer use experience. Additionally, the total administration time of the tool is less than 10 min for the majority of healthy people, which, considering the number of measures provided with self-scoring and self-reporting, is an extremely advantageous ratio. The tool can operate on both iOS and Windows as well as Android platforms, thanks to the flexibility of PsychoPy, which simplifies its application (Giaquinto et al., 2022).

## Figures and Tables

**Figure 1:** 

## Data Availability

The datasets presented in this study can be found in online repositories. The names of the repository/repositories and accession number(s) can be found below: the datasets analyzed for this study and SATURN materials can be found in the OSF repository at: https://osf.io/cdmt3/?view_only=44c8bc4977274e02969a2d7b80362caf.

## Footnotes

^1^ https://pavlovia.org/

^2^ https://opensciencetools.org/
