# Supplementary material for: Normative study of SATURN: a digital, self-administered, open-source cognitive assessment tool for Italians aged 50–80
Source: Front Psychol. 2024 Oct 30;15:1456619. doi: 10.3389/fpsyg.2024.1456619 (PMC11557479; doi:10.3389/fpsyg.2024.1456619)
Supplement: Supplementary file 1 [file Table_1.DOCX]

Supplementary Material

# Supplementary Tables

**Table S1** SATURN subdomain mean (±SD) accuracies and times on tasks as a function of age and education. Times are expressed in seconds.

| **Age - years** | | | | |
| --- | --- | --- | --- | --- |
| **Years of education** | | | |  |
| *SATURN Subdomain (accuracy)* | |  |  |  |
| *Attention* | 50-59 | 60-69 | 70-80 | Total |
| < 9 | 1.96(0.16) | 1.90(0.41) | 1.95(0.18) | 1.94(0.24) |
| 9-13 | 1.96(0.17) | 1.96(0.16) | 1.94(0.19) | 1.96(0.16) |
| > 13 | 1.98(0.11) | 1.94(0.20) | 1.83(0.31) | 1.95(0.18) |
| Total | 1.96(0.15) | 1.94(0.26) | 1.93(0.20) | 1.95(0.20) |
| *Incidental memory* | |  |  |  |
| < 9 | 0.91(0.17) | 0.82(0.22) | 0.84(0.23) | 0.87(0.20) |
| 9-13 | 0.90(0.18) | 0.83(0.22) | 0.89(0.22) | 0.88(0.20) |
| > 13 | 0.90(0.15) | 0.87(0.17) | 0.88(0.25) | 0.89(0.17) |
| Total | 0.90(0.17) | 0.84(0.20) | 0.85(0.23) | 0.87(0.19) |
| *Orientation* | |  |  |  |
| < 9 | 0.82(0.18) | 0.96(0.11) | 0.91(0.15) | 0.87(0.17) |
| 9-13 | 0.87(0.18) | 0.82(0.19) | 0.97(0.01) | 0.86(0.18) |
| > 13 | 0.76(0.15) | 0.75(0.17) | 0.67(0.00) | 0.75(0.16) |
| Total | 0.82(0.17) | 0.84(0.18) | 0.89(0.16) | 0.84(0.18) |
| *Recall memory* | |  |  |  |
| < 9 | 3.22(1.01) | 2.50(1.33) | 2.96(1.82) | 2.99(1.19) |
| 9-13 | 3.60(1.11) | 2.83(1.27) | 3.00(1.21) | 3.28(1.22) |
| > 13 | 3.81(1.05) | 3.48(0.96) | 2.63(1.41) | 3.55(1.10) |
| Total | 3.50(1.08) | 2.96(1.25) | 2.93(1.26) | 3.22(1.20) |
| *Math* | |  |  |  |
| < 9 | 1.29(0.46) | 1.12(0.55) | 1.24(0.46) | 1.24(0.48) |
| 9-13 | 1.24(0.50) | 1.34(0.40) | 1.50(0.00) | 1.30(0.44) |
| > 13 | 1.37(0.35) | 1.32(0.44) | 1.19(0.46) | 1.33(0.40) |
| Total | 1.29(0.46) | 1.27(0.47) | 1.28(0.43) | 1.28(0.45) |
| *Visuo-costructional abilities* | |  |  |  |
| < 9 | 0.75(0.25) | 0.69(0.27) | 0.67(0.28) | 0.71(0.26) |
| 9-13 | 0.80(0.23) | 0.84(0.18) | 0.75(0.21) | 0.81(0.21) |
| > 13 | 0.87(0.17) | 0.87(0.20) | 0.75(0.23) | 0.86(0.19) |
| Total | 0.80(0.23) | 0.81(0.23) | 0.69(0.27) | 0.78(0.24) |
| *Executive functions* | |  |  |  |
| < 9 | 1.43(0.34) | 1.12(0.51) | 1.37(0.32) | 1.35(0.39) |
| 9-13 | 1.44(0.33) | 1.31(0.42) | 1.56(0.30) | 1.41(0.36) |
| > 13 | 1.52(0.30) | 1.25(0.40) | 1.29(0.38) | 1.39(0.37) |
| Total | 1.45(0.33) | 1.23(0.44) | 1.40(0.33) | 1.37(0.38) |
| *SATURN Total time on task* | |  |  |  |
| < 9 | 417.95(213.33) | 430.10(140.09) | 360.61(207.39) | 399.89(200.30) |
| 9-13 | 379.63(181.15) | 439.26(199.01) | 452.89(370.95) | 406.84(214.52) |
| > 13 | 364.99(113.78) | 403.85(143.10) | 580.79(215.64) | 403.56(151.09) |
| Total | 391.19(182.60) | 424.74(164.85) | 402.19(250.72) | 403.09 (194.50) |
| *SATURN Subdomain (time on task)* | |  |  |  |
| *Attention* | |  |  |  |
| < 9 | 10.63(6.20) | 12.18(8.18) | 9.93(6.76) | 10.67(6.80) |
| 9-13 | 9.61(5.70) | 10.66(5.32) | 10.78(7.38) | 10.07(5.76) |
| > 13 | 8.38(2.67) | 8.93(3.47) | 13.37(6.95) | 9.14(3.86) |
| Total | 9.73(5.43) | 10.51(5.88) | 10.48(6.88) | 10.11 (5.88) |
| *Incidental memory* | |  |  |  |
| < 9 | 14.04(9.44) | 21.06(20.07) | 13.20(11.95) | 15.06(13.13) |
| 9-13 | 11.93(6.58) | 14.48(10.97) | 15.20(10.97) | 13.11(8.75) |
| > 13 | 10.59(4.90) | 13.19(6.11) | 19.16(10.95) | 12.55(6.66) |
| Total | 12.45(7.61) | 15.90(13.37) | 14.24(11.68) | 13.81(10.52) |
| *Orientation* | |  |  |  |
| < 9 | 9.06(6.67) | 7.85(3.73) | 6.87(3.49) | 8.06(5.28) |
| 9-13 | 6.55(2.53) | 7.42(3.05) | 7.97(5.52) | 6.99(3.15) |
| > 13 | 6.15(1.51) | 6.31(2.42) | 10.34(6.64) | 6.66(3.03) |
| Total | 7.44(4.67) | 7.17(3.11) | 7.46(4.40) | 7.37(4.21) |
| *Recall memory* | |  |  |  |
| < 9 | 94.01(59.30) | 84.05(60.94) | 93.73(72.08) | 92.04(64.07) |
| 9-13 | 90.49(61.26) | 104.92(77.18) | 108.25(99.46) | 97.08(71.14) |
| > 13 | 88.71(58.10) | 94.73(53.60) | 124.42(77.12) | 94.92(58.62) |
| Total | 91.45(59.46) | 95.59(65.30) | 99.82(77.40) | 94.42(4.21) |
| *Math* |  |  |  |  |
| < 9 | 25.25(22.26) | 18.06(8.87) | 15.15(10.95) | 20.31(17.39) |
| 9-13 | 23.41(27.42) | 23.57(17.32) | 23.55(30.41) | 23.48(24.76) |
| > 13 | 24.21(16.39) | 23.04(13.06) | 28.13(10.38) | 24.15(14.47) |
| Total | 24.31(23.16) | 21.84(13.99) | 18.12(16.39) | 22.82(19.65) |
| *Visuo-constructional abilities* | |  |  |  |
| < 9 | 20.85(15.08) | 19.76(13.30) | 14.68(11.59) | 18.46(13.80) |
| 9-13 | 19.36(15.00) | 22.82(17.24) | 24.99(25.02) | 21.09(16.99) |
| > 13 | 18.30(9.26) | 21.49(12.59) | 33.14(14.60) | 21.16(11.99) |
| Total | 19.70(13.89) | 21.51(14.61) | 18.62(16.16) | 18.98(14.60) |
| *Executive functions* | |  |  |  |
| < 9 | 29.61(14.57) | 35.86(13.50) | 29.29(15.05) | 30.67(14.66) |
| 9-13 | 26.87(9.83) | 32.75(10.19) | 31.91(22.67) | 29.32(12.15) |
| > 13 | 26.42(7.59) | 30.59(13.51) | 46.32(21.56) | 30.22(13.38) |
| Total | 27.83(11.55) | 32.90(12.39) | 31.72(17.89) | 30.11(13.53) |
| *Reading time* | |  |  |  |
| < 9 | 12.41(7.89) | 14.74(8.35) | 11.49(9.06) | 12.52(8.43) |
| 9-13 | 12.93(6.58) | 13.79(7.43) | 13.14(9.28) | 13.23(7.12) |
| > 13 | 12.09(4.50) | 14.05(5.75) | 18.14(4.66) | 13.53(5.33) |
| Total | 12.54(6.71) | 14.15(7.13) | 12.55(8.87) | 13.00(7.35) |

**Table S2** Multiple linear regression and beta unstandardised coefficients for the SATURN total accuracy, total time on tasks, and for the reading speed as a function of sociodemographic variables and help receiving during administration

| **Cognitive domains** | ***R*** | ***R^2^*** | ***F*** | ***P*** | **Beta (unstandardized coefficients)** | | |  |
| --- | --- | --- | --- | --- | --- | --- | --- | --- |
|  |  |  |  |  | **Sex** | **Age** | **Education** | **Help** |
| SATURN total accuracy | .256 | .065 | 5.562 | <.001 | ns | -0.061 | 0.105 | ns |
| SATURN total time | .182 | .033 | 2.739 | .029 | 68.453 | ns | ns | ns |
| Reading time | .129 | .017 | 1.339 | .255 | ns | ns | ns | ns |
